# Freestanding Ambulatory Surgery Centers and Patients Undergoing Outpatient Knee Arthroplasty

**DOI:** 10.1001/jamanetworkopen.2023.28343

**Published:** 2023-08-10

**Authors:** Charlotte M. Rajasingh, Laurence C. Baker, Sherry M. Wren

**Affiliations:** 1 Division of General Surgery, Department of Surgery, Stanford University School of Medicine, Stanford, California; 2 Department of Health Policy, Stanford University, Stanford, California

## Abstract

**Importance:**

In 2018, Medicare removed total knee arthroplasty from the list of inpatient-only procedures, resulting in a new pool of patients eligible for outpatient total knee arthroplasty. How this change was associated with the characteristics of patients undergoing outpatient knee arthroplasty at hospital-owned surgery centers (HOSCs) vs freestanding ambulatory surgery centers (FASCs) is unknown.

**Objectives:**

To describe the characteristics of patients undergoing outpatient, elective total and partial knee arthroplasty in 2017 and 2018 and to compare the cohorts receiving treatment at FASCs and HOSCs.

**Design, Setting, and Participants:**

This observational retrospective cohort study included 5657 patients having elective, outpatient partial and total knee arthroplasty in the Florida and Wisconsin State Ambulatory Surgery Databases in 2017 and 2018. Prior admissions were identified in the State Inpatient Database. Statistical analysis was performed from March to June 2022.

**Main Outcomes and Measures:**

Characteristics of patients undergoing surgery at a FASC vs a HOSC in 2017 and 2018 were compared.

**Results:**

A total of 5657 patients (mean [SD] age, 64.2 [9.9] years; 2907 women [51.4%]) were included in the study. Outpatient knee arthroplasties increased from 1910 in 2017 to 3747 in 2018 and were associated with an increase in total knee arthroplasties (474 in 2017 vs 2065 in 2018). The influx of patients undergoing outpatient knee arthroplasty was associated with an amplification of differences between the patients treated at FASCs and the patients treated at HOSCs. Patients with private payer insurance seen at FASCs increased from 63.4% in 2017 (550 of 867) to 72.7% in 2018 (1272 of 1749) (*P* < .001), while the percentage of patients with private payer insurance seen at HOSCs increased, but to a lesser extent (41.6% [427 of 1027] in 2017 vs 46.4% [625 of 1346] in 2018; *P* < .001). In 2017, the percentages of White patients seen at FASCs and HOSCs were similar (85.0% [737 of 867] vs 88.2% [906 of 1027], respectively); in 2018, the percentage of White patients seen at FASCs had increased and was significantly different from the percentage of White patients seen at HOSCs (90.6% [1585 of 1749] vs 87.9% [1183 of 1346]; *P* = .01). Both types of facilities saw an increase from 2017 to 2018 in the percentage of patients from communities of low social vulnerability, but this increase was greater for FASCs (FASCs: 6.7% [58 of 867] in 2017 vs 33.9% [593 of 1749] in 2018; HOSCs: 7.6% [78 of 1027] in 2017 vs 21.2% [285 of 1346] in 2018). Finally, while FASCs and HOSCs had cared for a similar portion of patients with prior admissions in 2017 (7.8% [68 of 867] vs 9.4% [97 of 1027], respectively; *P* = .25), in 2018, FASCs cared for fewer patients with prior admissions than HOSCs (4.0% [70 of 1749] vs 8.1% [109 of 1346]; *P* < .001).

**Conclusions:**

This study suggests that the increase in the number of patients undergoing outpatient knee arthroplasty in 2018 corresponded to FASCs treating a greater share of patients who were White, covered by private payer insurance, and healthier. These findings raise a concern that as more operations transition to the outpatient setting, variability in access to FASCs may increase, leaving hospital-owned centers to bear a greater share of the burden of caring for more vulnerable patients with more severe illness.

## Key Points

**Question:**

How did the population of patients undergoing outpatient knee arthroplasty at hospital-owned surgery centers and freestanding ambulatory surgery centers (FASCs) change after Medicare approved outpatient total knee arthroplasty in 2018?

**Findings:**

This cohort study found that the increased number of patients undergoing outpatient knee arthroplasty was associated with FASCs treating more patients who were White, insured by private payers, living in communities of low social vulnerability, and healthier.

**Meaning:**

These findings raise a concern that as operations transition to the outpatient setting, variability in access to FASCs may increase, leaving hospital-owned centers to care for a greater share of more vulnerable patients with more severe illness.

## Teaser

This cohort study describes the characteristics of patients undergoing outpatient, elective total and partial knee arthroplasty in 2017 and 2018 and compares the cohorts receiving treatment at freestanding ambulatory surgery centers and hospital-owned surgery centers.

## Introduction

Outpatient surgery constitutes most (>65%) surgical care in the United States and takes place at either freestanding ambulatory surgery centers (FASCs) or hospital-owned surgery centers (HOSCs).^1^ Before 2018, total knee arthroplasty (TKA) was most often performed in the inpatient setting, in contrast to partial knee arthroplasty, which was a common outpatient operation. In 2018, Medicare removed TKA from the inpatient-only list, and many private and non-Medicare insurance providers followed suit and more widely approved TKA as an outpatient procedure.^2^ Throughout 2018, Medicare continued to restrict outpatient TKA to only HOSCs, although many non-Medicare insurance providers allowed TKA at both FASCs and HOSCs.^3^ This change resulted in a large new pool of patients eligible for outpatient TKA.^2^

Patients from racial and ethnic minority groups, those with low income, and those covered by public payers less often have surgery at FASCs and, instead, are more likely to receive care at HOSCs.^4^ In the orthopedic literature, patients from racial and ethnic minority groups, those with low income, and those covered by public payers face challenges in accessing both elective and urgent orthopedic care, and previous studies suggest that variability in scheduling and patient triage is associated with delays of care.^5,6,7,8,9^ Knee arthroplasty is a highly discretionary procedure, making it sensitive to health care practitioner–introduced variability in who receives what care and where they receive it. Reimbursement for outpatient TKA is similar to reimbursement for partial knee arthroplasty, although the facility costs may be slightly higher for some TKAs.^10,11^ As a result, financial incentives do not necessarily favor one operation over the other and, again, health care practitioner selection may have a significant association with where patients receive care. The effect of the sudden increase in 2018 of eligible patients for outpatient knee arthroplasty on the patient mix at FASCs vs HOSCs is unknown.

We describe the characteristics of patients undergoing outpatient, elective TKA and partial knee arthroplasty in 2017 and 2018 and compare the cohorts treated at FASCs and HOSCs. We hypothesized that differences between the patients undergoing knee arthroplasty at FASCs vs HOSCs may increase with the larger pool of patients seeking care.

## Methods

### Data Source

We performed an observational retrospective cohort study of patients undergoing elective partial knee arthroplasty and TKA in the Florida and Wisconsin State Ambulatory Surgery Databases (SASDs), 2017 and 2018, from the Healthcare Cost and Utilization Project (HCUP) of the Agency for Healthcare Research and Quality. The SASD is a state-specific database that aggregates discharge records from ambulatory surgery. Each state has its own data collection organization, so there is variability across states in what data are reported and from what type of facility. We used Florida and Wisconsin because both states collect data from all HOSCs and FASCs in the state and report the type of facility. In addition, these states include a unique patient identifier for each record, which allows for linkage with the HCUP State Inpatient Databases (SIDs) and the HCUP State Emergency Department Databases (SEDDs), to identify hospital admissions and emergency department visits for a given patient. Because we were interested in prior admissions for patients, we used data from 2016 to 2018 for the SIDs and the SEDDs to ensure at least 1 year of prior data for all patients. Finally, based on reporting from the HCUP, the system for assigning unique patient identifiers within these states has remained consistent during these time periods, and as a result, patients can be tracked across data years in this sample.^12^ Our study followed the Strengthening the Reporting of Observational Studies in Epidemiology (STROBE) reporting guideline. Full review and patient consent were waived by the Stanford University institutional review board because all data were deidentified.

### Study Population

We identified all adult patients undergoing an elective, outpatient TKA or partial knee arthroplasty. Patients younger than 18 years were excluded. To ensure that we were including only patients undergoing outpatient, elective surgery, we excluded patients with a length of stay greater than 0 days, patients who had emergency department services billed at the encounter, patients who were admitted to an acute care facility after the ambulatory surgery, and patients with a missing unique patient identifier. Finally, Medicare patients undergoing outpatient TKA (*Current Procedural Terminology* code 27447) were selectively allowed at HOSCs. Therefore, while we report initially the total cohorts including Medicare patients undergoing TKA, we excluded these patients from further analysis because they were not eligible to have surgery at an FASC.

We compared patients whose operations were performed at an FASC with patients whose operation was performed at an HOSC. Freestanding centers have no associated American Hospital Association (AHA) identification number and do not contribute inpatient data to HCUP. An HOSC is associated with an AHA identification number, contributes to HCUP inpatient databases, or clearly provides alternative documentation indicating that the facility is hospital owned. An HOSC does not need to be in the same geographic location as the hospital with which it is associated.

### Patient-Level Covariates

The SASD records several covariates, including age, sex, race and ethnicity, primary payer, median household income of the patient’s zip code, and urban-rural classification of the patient’s zip code. The expected primary payer was categorized as private, Medicare, Medicaid, self-pay, or other (which included patients covered by Worker’s Compensation programs or medically indigent patients). Race and ethnicity are recorded in the Florida and Wisconsin SASDs based on reporting from hospital discharge records to the Florida Agency for Health Care Administration and the Wisconsin Department of Health Services, respectively. The HCUP reclassifies these variables into a standardized variable across the states that includes the categories of Asian or Pacific Islander, Black, Hispanic, Native American, White, and other (patients who identify with multiple racial groups or indicate that they do not identify with any of the listed groups); for the purposes of our study and because of the small numbers of patients in selected categories that would preclude reporting, we report the categories of Black, Hispanic, White, and other (which includes the HCUP categories of Asian or Pacific Islander, Native American, and other).

An additional covariate was the Social Vulnerability Index (SVI) for the patient’s home zip code. Although patient comorbidities can be used to control for underlying medical conditions that may be associated with both location of surgery and outcomes, differences in access to care and other social determinants of health may also be associated with location of surgery; these factors are only partially captured by demographic data in the SASD. The SVI is a metric measured by the Centers for Disease Control and Prevention. The SVI is a composite score based on 15 variables from US Census data. The SVI was initially validated as a factor associated with resilience against natural and human-caused disasters and an indicator of a region’s need for increased support during recovery. Subsequent work has identified a high SVI as a risk factor for decreased access to elective inpatient surgical care^13^ and worse postoperative outcomes.^14^ The SVI for each patient was identified by their county of residence as reported by Federal Information Processing System codes in the SASD. The scores are reported as a national percentile score ranging from 0 to 100.

The SASD does not report medical comorbidities, and the ambulatory surgery discharge records do not reliably include *International Statistical Classification of Diseases, Tenth Revision, Clinical Modification* (*ICD-10-CM*) diagnosis codes that capture comorbidities.^15^ To evaluate variability in patient comorbidity burden between the 2 types of facilities, we calculated Elixhauser comorbidity scores for the subset of patients who had a prior admission. We had 1 year of prior data available for all patients in the cohort (ie, SID inpatient admission data from 2016). *ICD-10-CM* codes from the prior admissions were extracted. The *comorbidity* package in R was used to identify the presence of Elixhauser comorbidities and calculate the Elixhauser comorbidity score with van Walraven weights.^16^ Of the comorbidities available, we report those that are included in the American College of Surgeons National Surgical Quality Improvement Program Risk Calculator^17^ and those that have been previously shown to be associated with readmission after knee arthroplasty.^18,19,20,21^

### Statistical Analysis

Statistical analysis was performed from March to June 2022. All statistical analysis was performed using R statistical software, version 4.1.2 and R Studio, version 2021.09.2 + 382 (R Group for Statistical Computing). The initial data extraction from the HCUP data files was performed using Stata, version 16 (StataCorp LP), and the HCUP provided Stata load files. Mean (SD) values are reported for variables that are normally distributed, while median (IQR) values are reported for variables that are nonnormal. The Wald *t* test was used to compare continuous variables, and the Pearson χ^2^ test was used to compare categorical variables. All tests were 2-sided, and *P* < .05 was considered statistically significant.

Unadjusted comparisons between the patient populations treated at FASCs vs HOSCs and between 2017 and 2018 were performed. Comorbidity comparisons are provided only for the subpopulation of patients with a prior inpatient admission. Postoperative outcomes included 30-day hospital admission and emergency department visit rates.

## Results

In the total cohort, 5657 patients (mean [SD] age, 64.2 [9.9] years; 2907 women [51.4%]) received outpatient TKAs and partial knee arthroplasties in 2017 and 2018 combined (Table 1). Most patients were White (5020 [88.7%]) and covered by private payer insurance (2874 [50.8%]) or Medicare (2450 [43.3%]).

**Table 1.** Characteristics of All Patients Undergoing Partial and Total Knee Arthroplasty

Characteristic	No. (%) of patients in 2017		*P* value	No. (%) of patients in 2018		*P* value
	HOSC (n = 1039)	FASC (n = 871)		HOSC (n = 1990)	FASC (n = 1757)	
Total knee arthroplasty	123 (11.8)	351 (40.3)	<.001	1065 (53.5)	1000 (56.9)	<.001
Partial knee arthroplasty	916 (88.2)	520 (59.7)	<.001	925 (46.5)	757 (43.1)	<.001
Sex						
Female	519 (50.0)	420 (48.2)	.48	1045 (52.5)	923 (52.5)	>.99
Male	520 (50.0)	451 (51.8)		945 (47.5)	834 (47.5)	
Age, mean (SD), y	65.8 (10.3)	62.6 (9.4)	<.001	66.8 (10.0)	61.2 (8.6)	<.001
Race and ethncity						
Black	36 (3.5)	44 (5.1)	.07	57 (2.9)	46 (2.6)	.16
Hispanic	42 (4.0)	33 (3.8)		76 (3.8)	45 (2.6)	
Other^a^	43 (4.1)	53 (6.1)		88 (4.4)	74 (4.2)	
White	918 (88.4)	741 (85.1)		1769 (88.9)	1592 (90.6)	
Primary payer						
Private	427 (41.1)	550 (63.1)	<.001	625 (31.4)	1272 (72.4)	<.001
Medicaid	17 (1.6)	≤10^b^		25 (1.3)	≤10^b^	
Medicare	552 (53.1)	286 (32.8)		1205 (60.6)	407 (23.2)	
Other	43 (4.1)	34 (3.9)		135 (6.8)	77 (4.4)	
Zip code income quartile^c^						
Bottom quartile ($1-$45 999)	307 (29.5)	164 (18.8)	<.001	391 (19.6)	241 (13.7)	<.001
Second quartile ($46 000-$58 999)	334 (32.1)	279 (32.0)		738 (37.1)	525 (29.9)	
Third quartile ($59 000-$78 999)	274 (26.4)	278 (31.9)		593 (29.8)	600 (34.1)	
Top quartile (≥$79 000)	113 (10.9)	138 (15.8)		252 (12.7)	372 (21.2)	
Social Vulnerability Index quartile^d^						
Bottom quartile (least vulnerable)	80 (7.7)	58 (6.7)	.007	568 (28.5)	593 (33.8)	<.001
Second quartile	277 (26.7)	217 (24.9)		417 (21.0)	343 (19.5)	
Third quartile	574 (55.2)	538 (61.8)		818 (41.1)	741 (42.2)	
Top quartile (most vulnerable)	107 (10.3)	58 (6.7)		186 (9.3)	80 (4.6)	
Admitted within prior 1 year	98 (9.4)	68 (7.8)	.24	178 (8.9)	70 (4.0)	<.001

Abbreviations: FASC, freestanding ambulatory surgery center; HOSC, hospital-owned surgery center.

^a^ Asian or Pacific Islander, Native American, and other.

^b^ Per the Healthcare Cost and Utilization Project data use agreement, no cell sizes less than or equal to 10 are reported.

^c^ Ranges reported are for 2018.

^d^ Data were not available for 1 patient.

There was a substantial increase in the number of outpatient TKAs and partial knee arthroplasties performed at HOSCs and FASCs combined, from 1910 in 2017 to 3747 in 2018 (Figure, A). This increase was predominantly associated with an increase in the number of outpatient TKAs at both facilities such that outpatient TKA was performed more frequently than partial knee arthroplasty in 2018 (2065 vs 1682) (Table 1). After accounting for an increase in the number of reporting facilities, there was still an increase in the mean number of knee arthroplasties performed at both facility types (mean partial knee arthroplasty or TKA per facility in 2017 vs 2018: 15 vs 27 at FASCs; 11 vs 14 at HOSCs ).

**Figure.**
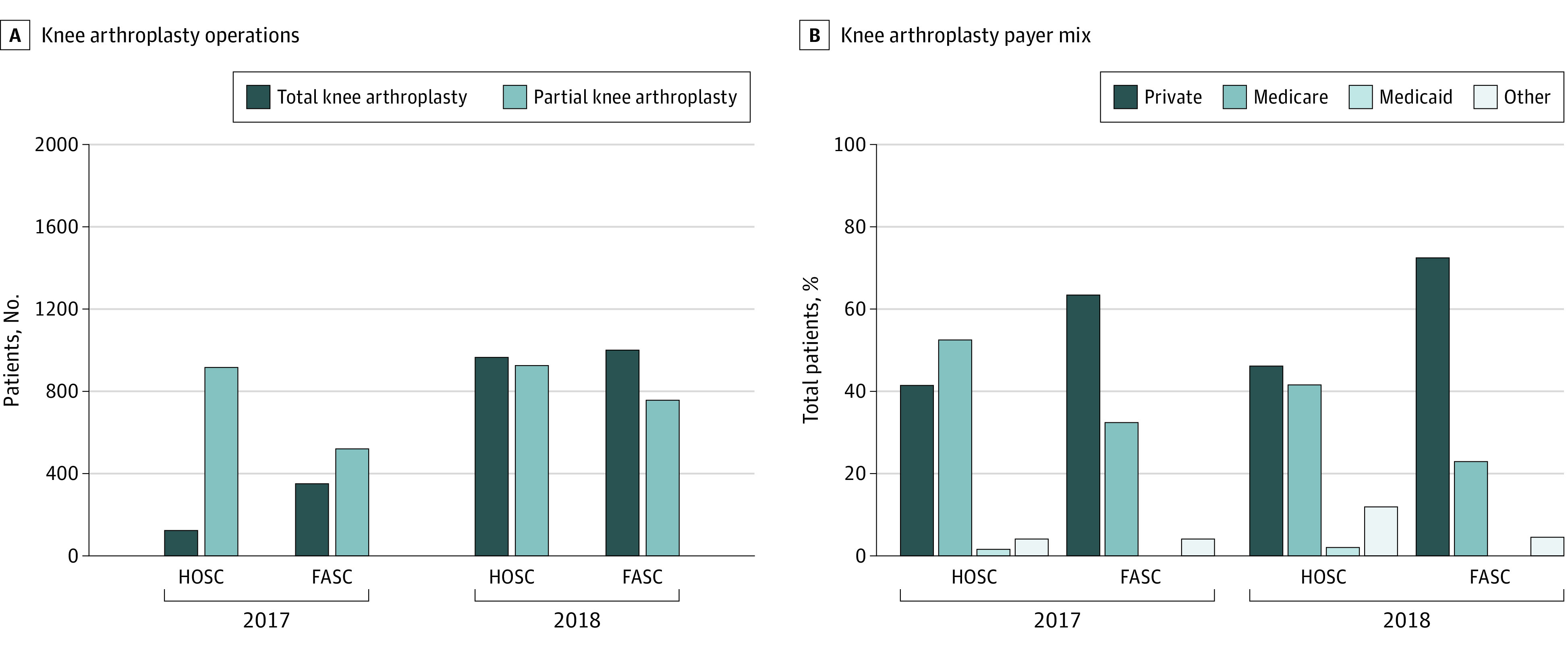
Number of Total and Partial Knee Arthroplasty Cases in 2017 and 2018 and Payer Mix for All Knee Arthroplasty Cases in the Population of Patients Eligible to Have Surgery at Hospital-Owned Surgery Centers (HOSCs) and Freestanding Ambulatory Surgery Centers (FASCs)

Consistent with the fact that Medicare patients having outpatient TKA were eligible for surgery only at an HOSC, the percentage of Medicare patients in HOSCs increased from 2017 to 2018 (552 of 1039 [53.1%] vs 1205 of 1990 [60.6%]) (Table 1). This corresponded to HOSCs seeing older patients and more patients previously admitted to the hospital than FASCs in 2018.

To evaluate how patients eligible for surgery at both FASCs and HOSCs were distributed between the 2 facility types, we excluded patients covered by Medicare who underwent TKA from further analysis because these patients could only have surgery at HOSCs. Overall, the sudden influx of patients undergoing TKA was associated with an increase in differences between patients seen at FASCs and HOSCs in 2018 compared with 2017. Among patients eligible for surgery at both FASCs and HOSCs, FASCs saw significantly more patients with private payer insurance, while HOSCs saw more patients with Medicare (Figure, B). The percentage of patients with private payer insurance seen at FASCs increased from 63.4% in 2017 (550 of 867) to 72.7% in 2018 (1272 of 1749) (*P* < .001), while the percentage of patients with private payer insurance seen at HOSCs increased, but to a lesser extent (41.6% [427 of 1027] in 2017 vs 46.4% [625 of 1346] in 2018; *P* < .001) (Table 2).

**Table 2.** Characteristics of Patients Eligible for Surgery at Both FASCs and HOSCs

Characteristic	No. (%) of patients in 2017		*P* value	No. (%) of patients in 2018		*P* value
	HOSC (n = 1027)	FASC (n = 867)		HOSC (n = 1346)	FASC (n = 1749)	
Total knee arthroplasty	111 (10.8)	347 (40.0)	<.001	421 (31.3)	992 (56.7)	<.001
Partial knee arthroplasty	916 (89.2)	520 (60.0)	<.001	925 (68.7)	757 (43.3)	<.001
Sex						
Female	512 (49,9)	416 (48.0)	.44	671 (49.9)	917 (52.4)	.17
Male	515 (50.1)	451 (52.0)		675 (50.1)	832 (47.6)	
Age, mean (SD), y	65.8 (10.3)	62.6 (9.4)	<.001	64.7 (10.7)	61.2 (8.6)	<.001
Race and ethnicity						
Black	36 (3.5)	44 (5.1)	.08	49 (3.6)	46 (2.6)	.01
Hispanic	42 (4.1)	33 (3.8)		60 (4.5)	45 (2.6)	
Other^a^	43 (4.2)	53 (6.1)		54 (4.0)	73 (4.2)	
White	906 (88.2)	737 (85.0)		1183 (87.9)	1585 (90.6)	
Primary payer						
Private	427 (41.6)	550 (63.4)	<.001	625 (46.4)	1272 (72.7)	<.001
Medicaid	17 (1.7)	≤10^b^		25 (1.9)	≤10^b^	
Medicare	540 (52.6)	282 (32.5)		561 (41.7)	399 (22.8)	
Other	43 (4.2)	34 (3.9)		135 (10.0)	77 (4.4)	
Zip code income quartile^c^						
Bottom quartile ($1-$45 999)	304 (29.6)	163 (18.8)	<.001	281 (20.9)	241 (13.8)	<.001
Second quartile ($46 000-$58 999)	330 (32.1)	278 (32.1)		525 (39.0)	521 (29.8)	
Third quartile ($59 000-$78 999)	269 (26.2)	277 (31.9)		380 (28.2)	597 (34.1)	
Top quartile (≥$79 000)	113 (11.0)	137 (15.8)		151 (11.2)	371 (21.2)	
Social Vulnerability Index quartile						
Bottom quartile (least vulnerable)	78 (7.6)	58 (6.7)	.84	285 (21.2)	593 (33.9)	<.001
Second quartile	276 (26.9)	215 (24.8)		316 (23.5)	340 (19.4)	
Third quartile	566 (55.1)	536 (61.8)		612 (45.5)	736 (42.1)	
Top quartile (most vulnerable)	106 (10.3)	58 (6.7)		132 (9.8)	80 (4.6)	
Admitted within prior 1 year	97 (9.4)	68 (7.8)	.25	109 (8.1)	70 (4.0)	<.001

Abbreviations: FASC, freestanding ambulatory surgery center; HOSC, hospital-owned surgery center.

^a^ Asian or Pacific Islander, Native American, and other.

^b^ Per the Healthcare Cost and Utilization Project data use agreement, no cell sizes less than or equal to 10 are reported.

^c^ Ranges reported are for 2018.

In 2017, the percentage of White patients seen at FASCs vs HOSCs was not significantly different (85.0% [737 of 867] vs 88.2% [906 of 1027]); in 2018, the percentage of White patients seen at FASCs had increased compared with 2017 and was then significantly different from the percentage of White patients seen at HOSCs (90.6% [1585 of 1749] vs 87.9% [1183 of 1346]) (Table 2). In 2018 compared with 2017, a larger share of patients receiving care at FASCs lived in high-income zip code regions (137 of 867 [15.8%] in 2017; 371 of 1749 [21.2%] in 2018), while the percentage of patients receiving care at HOSCs living in zip code regions with the highest income quartile was relatively similar between 2017 and 2018 (113 of 1027 [11.0%] vs 151 of 1346 [11.2%]). Both types of facilities saw an increase from 2017 to 2018 in the percentage of patients from communities of low social vulnerability, but this increase was greater for FASCs (FASCs: 6.7% [58 of 867] in 2017 vs 33.9% [593 of 1749] in 2018; HOSCs: 7.6% [78 of 1027] in 2017 vs 21.2% [285 of 1346] in 2018).

Finally, while FASCs and HOSCs had cared for a similar portion of patients with prior admissions in 2017 (7.8% [68 of 867] vs 9.4% [97 of 1027], respectively; *P* = .25), in 2018 FASCs compared with HOSCs cared for fewer patients with prior admissions (4.0% [70 of 1749] vs 8.1% [109 of 1346]; *P* < .001) (Table 2).

Comorbidity data are very limited because we were able to extract comorbidities and calculate comorbidity scores only for the percentage of patients who had been admitted to the hospital in the preceding 1 year (n = 344). In this subpopulation, the median Elixhauser comorbidity score in 2017 was 0 (IQR, 0.0-5.0) at HOSCs and 0 (IQR, –0.3 to 5.0) at FASCs; in 2018, the median Elixhauser comorbidity score was 0 (IQR, 0.0-6.0) at HOSCs and 0 (IQR, –2.8 to 4.0) at FASCs (Table 3). The mean scores were not significantly different. Rates of individual comorbidities were generally similar between FASCs and HOSCs, which did not change substantially from 2017 to 2018.

**Table 3.** Patient Comorbidities in Patients With a Prior Hospital Admission^a^

Characteristic	No. (%) of patients in 2017		*P* value	No. (%) of patients in 2018		*P* value
	HOSC (n = 97)	FASC (n = 68)		HOSC (n = 109)	FASC (n = 70)	
Age, mean (SD), y	66.8 (9.6)	62.1 (10.0)	.003	65.6 (11.4)	59.0 (8.5)	<.001
Elixhauser comorbidity score						
Median (IQR)	0 (0.0 to 5.0)	0 (–0.3 to 5.0)	.95	0 (0.0 to 6.0)	0 (–2.8 to 4.0)	.17
Mean (SD)	2.2 (6.4)	2.0 (5.8)	.79	3.4 (7.1)	1.9 (6.4)	.14
Hypertension	73 (75.3)	48 (70.6)	.63	71 (65.1)	40 (57.1)	.36
Chronic pulmonary disease	16 (16.5)	≤10^b^	.53	18 (16.5)	14 (20.0)	.69
Diabetes	17 (17.5)	12 (17.6)	>.99	20 (18.3)	20 (28.6)	.16
Obesity	20 (20.6)	21 (30.9)	.19	34 (31.2)	25 (35.7)	.64
Mental health disorder^c^	25 (25.8)	11 (16.2)	.20	24 (22.0)	17 (24.3)	.87

Abbreviations: FASC, freestanding ambulatory surgery center; HOSC, hospital-owned surgery center.

^a^ Peripheral vascular disease, kidney failure, liver disease, congestive heart failure, and rheumatoid arthritis or collagen vascular disease were also examined, but not reported because there were 10 patients or fewer for at least 2 cells.

^b^ Per the Healthcare Cost and Utilization Project data use agreement, no cell sizes less than or equal to 10 are reported.

^c^ Includes alcohol abuse, drug abuse, psychotic disorders, and depression.

Postoperative outcomes including emergency department visits and hospital admissions were examined (Table 4). Hospital admission rates did not differ between FASCs and HOSCs in 2017 or in 2018. Emergency department visit rates were statistically similar in 2017, but in 2018, they were significantly lower at FASCs than HOSCs (4.0% [70 of 1749] vs 6.1% [82 of 1346]; *P* = .01).

**Table 4.** Postoperative ED Visits and Hospital Admissions

Characteristic^a^	No. (%) of patients in 2017		*P* value	No. (%) of patients in 2018		*P* value
	HOSC (n = 1027)	FASC (n = 867)		HOSC (n = 1346)	FASC (n = 1749)	
ED visit within 3 d	19 (1.9)	≤10^b^	.21	23 (1.7)	23 (1.3)	.46
ED visit within 30 d	60 (5.8)	38 (4.4)	.19	82 (6.1)	70 (4.0)	.01
Hospital admission within 30 d	23 (2.2)	≤10^b^	.10	28 (2.1)	28 (1.6)	.39

Abbreviations: ED, emergency department; FASC, freestanding ambulatory surgery center; HOSC, hospital-owned surgery center.

^a^ Hospital admissions within 3 days are not reported as all cells contained 10 patients or fewer.

^b^ Per the Healthcare Cost and Utilization Project data use agreement, no cell sizes less than or equal to 10 are reported.

## Discussion

From 2017 to 2018, there was a significant increase in the number of patients undergoing outpatient TKA coincident with changes in the Medicare reimbursement rules surrounding TKA. This increase manifested as an increase in the number of TKAs performed at both HOSCs and FASCs, and therefore, an increase in the total pool of patients undergoing TKA and partial knee arthroplasty.

The increase in patients undergoing outpatient knee arthroplasty corresponded to a shift in the demographic profile of patients having knee arthroplasty surgery at FASCs vs HOSCs. Although FASCs had cared for more wealthy, healthy, and White patients than HOSCs in 2017, this difference became more pronounced in 2018. The payer distribution between FASCs and HOSCs similarly shifted from 2017 to 2018, with FASCs capturing a larger share of privately insured patients in 2018 compared with HOSCs. Finally, where HOSCs and FASCs had cared for a comparable percentage of patients with a history of hospital admission in 2017, FASCs cared for significantly fewer of these patients in 2018.

Our findings add to the existing body of work on access to ambulatory surgery centers and suggest that the increase in the pool of patients having ambulatory surgery may be associated with an increase in the differences in the types of patients seen at FASCs and HOSCs. There are several potential explanations for our findings. Because FASCs are independent of a hospital and potentially more limited in their ability to care for sick patients, directing healthier patients toward FASCs may be appropriate. Demographic variability between FASC and HOSC patients may then be secondary to population-level differences in the prevalence of common, comorbid diseases.^22^ However, in our findings, the shift in demographic characteristics from 2017 to 2018 when the total pool of patients was larger suggests that patient selection may be sensitive to the number of patients seeking care. This possibility is underscored by the fact that the share of patients previously admitted to the hospital was similar at FASCs and HOSCs in 2017 but significantly different in 2018 and that among that population of patients, HOSC patients did not have significantly higher rates of individual comorbid diseases, such as chronic pulmonary disease or congestive heart failure. Given the existing evidence that demographic characteristics are independently associated with location of surgery and that patients of certain demographic profiles struggle to access outpatient orthopedic care,^4,5,9,23^ our findings further highlight existing variability in access to FASCs and the potential for these differences to be exacerbated as outpatient surgical volume increases.

The potential consequences of variability in access to FASCs are context specific and perspective specific. For individuals, cost and experience may vary between facility type and may be variable based on the type of procedure.^11,24^ A Medicare enrollee, for example, pays out of pocket nearly twice as much for a laparoscopic cholecystectomy at an HOSC than at an FASC but pays more for a partial knee arthroplasty at an FASC than at an HOSC.^11^ Not being able to access certain facilities may put a financial burden on select groups of patients depending on the terms of their insurance coverage.

Similarly, although there is some debate about whether FASCs are truly cost saving for insurance providers, HOSCs are almost universally reimbursed at higher rates than FASCs.^3,11,25^ Reduced access to FASCs for patients covered by public payers theoretically shifts the burden of higher-cost operations at HOSCs onto the public payer programs. Because lower reimbursement rates for FASCs has been associated with a slowdown in the growth of FASCs,^26^ this in itself may further limit access to FASCs. Finally, for many hospital systems, HOSCs are revenue generating. As others have argued,^4^ if HOSCs are increasingly left to care for a less favorable payer mix, this may negatively affect a hospital system’s ability to provide other essential but traditionally less profitable services.

### Limitations

There are several limitations to this analysis. We lacked patient-level comorbidity data for many patients, and patient comorbidities likely play an important role in dictating what types of patients are seen at FASCs or HOSCs. The subset of patients for whom comorbidity data were extracted from prior admissions likely skewed toward sicker patients and, therefore it is notable that even in this subset, there was minimal difference in the comorbidity profiles. However, without complete comorbidity data on all patients, our conclusions about the patterns observed in this study are limited. We are similarly limited by the lack of granular clinical data and geographic data on access to FASCs and HOSCs, both of which may be associated with patient referral patterns.

Finally, this study is limited to data from Florida and Wisconsin. Many states do not collect data from FASCs. For example, since 2007, physician-owned FASCs in California have no longer been obligated to report data to California’s Office of Statewide Health Planning and Development, which contributes data to the SASD; the subsequent number of FASC-reported cases decreased by more than 90%.^27^ We are not aware of other all-payer data sources that capture granular clinical data on ambulatory surgery at HOSCs and FASCs, but we hope that this work and other investigations into this topic may highlight the need for improved data collection.

## Conclusion

This cohort study found that the substantial increase in the number of patients undergoing outpatient knee arthroplasty in 2018 corresponded to FASCs seeing a greater share of patients who were White, covered by private payer insurance, living in communities of low social vulnerability, and healthier. These findings raise a concern that as more operations transition to the outpatient setting, variability in access to FASCs may increase, leaving HOSCs to bear a greater share of the burden of caring for more vulnerable patients with more severe illness.

## Notes

Supplement.
Data Sharing Statement
