# Supplementary material for: Freestanding Ambulatory Surgery Centers and Patients Undergoing Outpatient Knee Arthroplasty
Source: JAMA Netw Open. 2023 Aug 10;6(8):e2328343. doi: 10.1001/jamanetworkopen.2023.28343 (PMC10415959; doi:10.1001/jamanetworkopen.2023.28343)
Supplement: Supplement. — Data Sharing Statement [file jamanetwopen-e2328343-s001.pdf]

Data Sharing Statement

Rajasingh. Freestanding Ambulatory Surgery Centers and Patients Undergoing Outpatient Knee Arthroplasty. *JAMA Netw Open*. Published August 10, 2023. doi:10.1001/jamanetworkopen.2023.28343

Data
Data available: No

Additional Information
Explanation for why data not available: The Data Use Agreement signed at the time of purchase precludes sharing of the data. However, this data is publicly available through HCUP, AHRQ.
